# Elevated Glucagon-like Peptide-1 and a Th2 Shift May Support Reduced Prevalence of Thoracic Aortic Aneurysm in Patients with Diabetes

**DOI:** 10.3390/jcdd8110143

**Published:** 2021-10-28

**Authors:** Stelia Ntika, Harshitha Jois, Karin Lång, Christian Olsson, Anders Franco-Cereceda, Hanna M. Björck, Camilla Krizhanovskii

**Affiliations:** 1 Department of Research, Södertälje Hospital, SE-152 86 Södertälje, Sweden; hjoi@food.dtu.dk; 2 Cardiothoracic Surgery Unit, Department of Molecular Medicine and Surgery, Karolinska Institutet, SE-171 76 Stockholm, Sweden; christian.olsson@ki.se (C.O.); anders.franco-cereceda@ki.se (A.F.-C.); 3 Center for Molecular Medicine, Cardiovascular Medicine Unit, Department of Medicine, Karolinska Institutet, Karolinska University Hospital Solna, SE-171 64 Stockholm, Sweden; karin.lang@ki.se (K.L.); hanna.bjorck@ki.se (H.M.B.); 4 Department of Clinical Science and Education, Karolinska Institutet, Stockholm South Hospital, SE-118 83 Stockholm, Sweden

**Keywords:** aneurysm, type 2 diabetes, glucagon-like peptide-1, inflammation, cytokines

## Abstract

Glucagon-like peptide-1 (GLP-1) regulates processes involved in the pathophysiology of thoracic aortic aneurysms (TAAs), including inflammation, while protecting against aortic aneurysms in animal models. Type 2 diabetes (T2D) involves altered GLP-1 signaling due to pathology and/or therapy and is associated with reduced prevalence of TAAs. We aimed to assess whether T2D alters the inflammatory profile/proteolytic activity, possible correlations to elevated fasting GLP-1 (F-GLP-1), and its relevance for TAA. F-GLP-1, pro-inflammatory T helper 1 (Th1) cytokines, Th2 cytokines, C-reactive protein, and matrix metalloproteinase-2 activity (MMP-2) were analyzed in surgical patients with aortic valve pathology with/without T2D and without T2D but with TAA. Patients with T2D displayed an increase in the relative systemic expression of interleukin 6 and tumor necrosis factor α and a clear trend towards reduced levels of interferon γ (IFNγ). In addition, a positive association between GLP-1 and the plasma interleukin 4 (IL-4)/IFNγ ratio was detected. TAA was associated with significantly lower plasma levels of the Th2 cytokines IL-4 and interleukin 5. Plasma MMP-2 activity did not differ between groups. We conclude that T2D involved a Th2 shift, which associates with elevated F-GLP-1 and may—considering Th1 bias in TAA—contribute to reduced prevalence of TAA in T2D.

## 1. Introduction

Thoracic aortic aneurysms (TAA) are balloon-like dilations of the aorta above the diaphragm, caused by a weakening of the aortic wall. When the aneurysm reaches a certain size or if inflammation/cell death in the area causes the layers of the aortic wall to degenerate, the aorta may rupture, resulting in life-threatening internal bleeding. Approximately 25% of aortic aneurysms (AA) are TAAs, the remaining are abdominal aortic aneurysms (AAAs). Smoking, valve pathology, and genetic conditions, such as Marfan syndrome, are risk factors for TAAs [1].

However, type 2 diabetes (T2D) is associated with reduced prevalence of TAA [2,3]. One of the largest studies to date, found an overall pooled risk reduction of 15% for TAAs and AAAs among patients with T2D, as compared with controls [4]. Another longitudinal observational study of nearly three million individuals found a relative risk reduction of 28% for AAs (including both TAAs and AAAs) and 44% for TAAs [5]. None of these studies considered anti-diabetic therapy when assessing the prevalence of TAA in T2D, and the risk reduction conferred by T2D may thus involve pathophysiological mechanisms and/or effects of antidiabetic therapy. Unraveling the factors/mechanisms contributing to the protective effect of T2D may contribute to a first future pharmacological intervention in TAA, which is increasing in prevalence and is a critical condition, despite modern diagnostic tools and current surgical/endovascular repair.

Interestingly, glucagon-like-peptide-1-based (GLP-1-based) antidiabetic therapy protects from AAA formation in animal models [6], suggesting a role for GLP-1 in the reduced prevalence of AA in T2D. GLP-1 is produced in the intestine and the nucleus of the tractus solitarii in the brainstem [7]. The effects of GLP-1 and its analogs on target tissues, such as the pancreas and the central nervous system (CNS), include improved glucose tolerance [8]. The active forms of GLP-1 (7-36 amide and 7-37) are rapidly degraded by dipeptidyl peptidase-4 (DPP-4), and efferent signals contribute to the effects exerted by the native peptide [9], while stable GLP-1 analogs and DPP-4 inhibitors are used in T2D therapy. However, the GLP-1 receptor is widely expressed, and direct effects of the peptide on vascular tissue have been demonstrated. Further, although GLP-1 secretion is stimulated by nutrient intake and fasting levels are low, increased fasting plasma GLP-1 (F-GLP-1) has been detected in T2D [10] and is implicated in higher rates of energy expenditure, fat oxidation, and cardioprotective effects [11].

GLP-1 and/or GLP-1 analogs have been indicated to regulate mechanisms involved in TAA formation [2], including inflammatory responses and proteolytic activity.

The Th1 subtype of T cells secretes pro-inflammatory cytokines, such as interferon γ (IFN-γ) and tumor necrosis factor α (TNF-α). The Th2 subtype of T cells secretes anti-inflammatory cytokines such as interleukin-4 (IL-4) and interleukin-5 (IL-5) and is associated with anti-inflammatory processes through regulation of the responses to Th1 cytokines [12,13]. The balance of Th1/Th2 responses governs the induced proteolytic activity and is important in the etiology/pathology of cardiovascular diseases such as TAAs. Specifically, a Th1 bias of immune responses is indicated in TAA formation and progression [14,15,16]. However, it is not known whether or how F-GLP-1 levels are associated with the differential expression of Th1/Th2 cytokines and/or altered proteolytic activity.

We hypothesized that GLP-1 signaling contributes to the reduced prevalence/progression of TAAs in T2D, by favorably regulating inflammatory responses and proteolytic activity. Consequently, the aim of this study was to determine potential differences in systemic inflammation and proteolytic activity between non-T2D and T2D patients, and whether such changes are associated with elevated F-GLP-1 in T2D. Furthermore, to assess the possible relevance of such changes/associations, the same parameters were assessed in patients with TAAs.

## 2. Materials and Methods

### 2.1. Patient Characteristics

**Figure 1:**
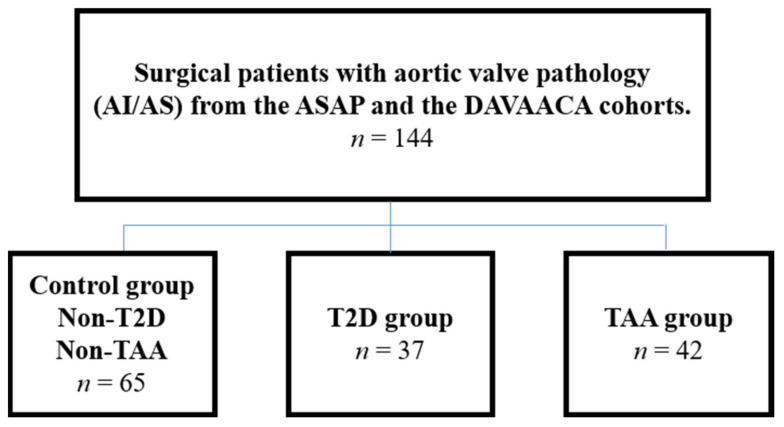
Flowchart of study groups.

Inclusion criteria: surgical patients with aortic valve pathology (aortic insufficiency/aortic stenosis) and included in the Advanced Study of Aortic Pathology (ASAP)/Disease of the Aortic Valve, Ascending Aorta and Coronary Arteries (DAVAACA) cohorts [17]. Intraoperative echocardiography was used for ascending aortic measurements, and the aorta was classified as normal if <40 mm and dilated if >45 mm in diameter. Exclusion criteria: Ascending aortic diameter dimensions between 40 and 45 mm, Marfan syndrome, bicuspid/monocuspid valves, and atherosclerosis. Patients were divided into three groups, depending on the presence of T2D/TAAs—generating a control group of non-T2D patients without TAA, a T2D group without TAA, and a third group of non-T2D patients with TAA (Flowchart of study groups, Figure 1). One-hundred and fifty-two plasma samples were obtained from patients meeting the inclusion criteria, and eight out of these 152 patients had both T2D and TAAs. These eight patients were excluded due to the difficulty in discriminating between changes resulting from the TAA formation and changes that may identify a “subset” of T2D patients in whom TAA formation is occurring.

T2D diagnosis was self-reported, based on the criteria of the American Diabetes Association (2014). Similar to other studies investigating the reduced risk of TAAs and AAAs in T2D patients, all T2D patients were included, irrespective of T2D duration, hemoglobin A1c (HbA1c) levels, and medication. Patient characteristics, including age, anthropometry, medications, aortic valve pathology, and gender distribution, are shown in the Supplementary Materials, Table S1. Ethical permission was obtained from the Stockholm Regional Ethical Committee (Dnr: 2006/78431/1; approved: 15 September 2006) and (Dnr: 2012/1633-31/4; approved 24 October 2012).

### 2.2. F-GLP-1 Levels

The F-GLP-1 levels were analyzed in all patients (*n* = 144). Plasma was collected after at least eight hours of fasting. A DPP-4 inhibitor (10 µL/mL, Cat. No.: DPP4, Millipore, Burlington, Massachusetts) was added to a subset of plasma samples immediately after sampling for the analysis of active GLP-1. Total GLP-1 (7-36 amide and 9-36) and active GLP-1 (7-36 amide and 7-37) were measured with enzyme-linked immunosorbent assay (ELISA) kits (Cat. No.: EZGLP1T-36K, and Cat. No.: EZGLPHS-35K, Millipore, Burlington, Massachusetts), according to the manufacturer’s instructions.

### 2.3. Cytokines and High-Sensitivity C-Reactive Protein (hsCRP)

Seven cytokines were analyzed, namely TNF-α, interleukin-6 (IL-6), interleukin 1β (IL-1β), IFN-γ, interleukin-12p70, IL-4, and IL-5, as well as the inflammatory biomarker hsCRP. Cytokines were measured with an MSD multiplex ELISA Kit (Cat. No.: K15067L-1, Mesoscale Discovery, Rockville, Maryland), according to the manufacturer’s instructions. The hsCRP was measured during the routine analysis by an ultrasensitive CRP kit (Orion Diagnostica, Espoo, Finland).

### 2.4. Matrix Metalloproteinase-2 (MMP-2) Activity

MMP-2 activity was measured with a human MMP-2 activity assay (Cat. No.: QZBmmp2Hv2, Quickzyme Biosciences, Leiden, The Netherlands), according to the manufacturer’s instructions. Briefly, the assay is based on the release of color from a chromogenic peptide substrate upon the activation of a pro-enzyme. The assay measures both active MMP-2 and pro-MMP-2, which is activated on the plate by p-aminophenyl mercuric acetate (APMA) solution. For this study, only endogenous active MMP-2 was measured.

### 2.5. In Vitro GLP-1 Secretion Studies

GLUTag enteroendocrine L-cells, graciously donated by Dr. Neil Portwood, Karolinska Institutet, Solna, Sweden, and originally from Dr. Daniel J. Drucker, Mount Sinai Hospital, Samuel Lunenfeld Research Institute, University of Toronto, Canada, were used as a model for the in vitro studies. Culture conditions and detailed protocol (Supplementary Materials File).

### 2.6. Statistical Analysis

The unpaired Student two-tailed *t*-test was used for two-sample comparisons. Comparisons between multiple groups were performed with one-way ANOVA. Outliers were identified with the robust regression and outlier removal (ROUT) test in GraphPad, with Q = 1%, where the value defines how aggressive the test is. Correlations were assessed using the Pearson correlation coefficients (95% CI). Linear regression analysis was used for the graphs, which were plotted with GraphPad Prism 6 (GraphPad Software, San Diego, California), and the data are represented as ± SEM. *p* < 0.05 was considered significant for a two-sided hypothesis. Analysis of covariance (ANCOVA) was performed using the R studio software version 4.0.3.

## 3. Results

### 3.1. Patient Characteristics

Patients were divided into three groups, depending on the presence of T2D/TAAs—generating a control group of non-T2D patients without TAA, a T2D group without TAA, and a third group of non-T2D patients with TAA (flowchart of study groups, Figure 1). Patient characteristics, including age, anthropometry, medications, aortic valve pathology, and gender distribution, are shown in the vs, Table S1.

### 3.2. T2D Is Associated with an Increase in IL-6/TNF-α Ratio

**Figure 2:**
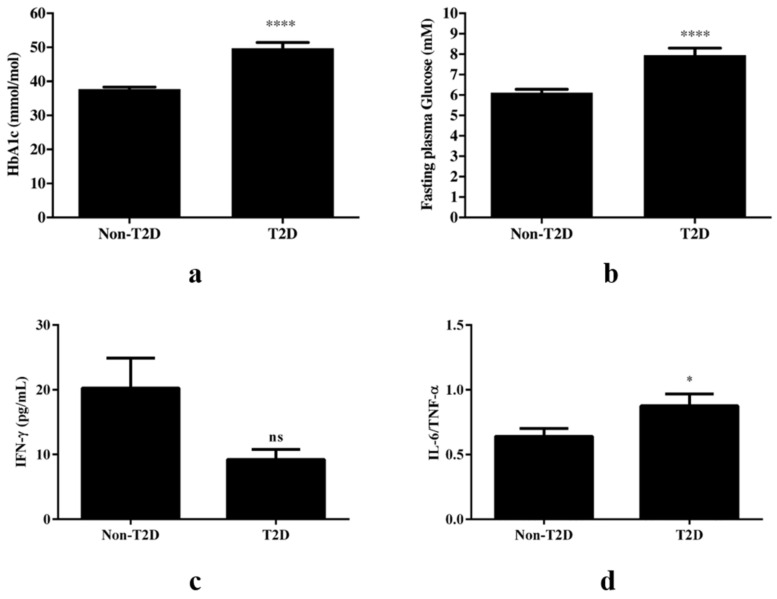
T2D is associated with an increase in IL-6/TNF-α ratio. (**a**) HbA1c (*n* = 63 for non-T2D, *n* = 36 for T2D) and (**b**) fasting plasma glucose (*n* = 62 for non-T2D, *n* = 35 for T2D) levels were higher in T2D patients, *p* < 0.0001 for both. (**c**) A clear trend towards reduced IFN-γ levels was observed in patients with T2D when compared to the non-T2D group (*p* = 0.06) and (**d**) Fasting plasma IL-6/TNF-α ratio (*n* = 49 for non-T2D, *n* = 33 for T2D) was significantly increased in T2D compared with the non-T2D group (*p* < 0.05). Comparisons between groups were done with an unpaired *t*-test. ns = no significant; * *p* < 0.05; **** *p* < 0.0001.

Reported T2D was, as expected, characterized by HbA1c levels ≥48 mmol/mol (49.7 ± 1.7, Figure 2a) and fasting plasma glucose ≥7 mM (8.0 ± 0.3, Figure 2b), indicative of T2D. No significant differences in systolic blood pressure, diastolic blood pressure, hsCRP, or any of the cytokines analyzed were detected in association with T2D (Supplementary Materials, Table S2). However, there was a clear trend towards reduced plasma levels of IFN-γ in the T2D group, as compared to the control group (7.96 ± 0.86 vs. 20.02 ± 4.63, *p* = 0.06, Figure 2c). The relative systemic expression of two early response cytokines; the multifunctional cytokine with anti-inflammatory and Th2-polarizing properties IL-6 and the pro-inflammatory cytokine TNF-α. (IL-6/TNF-α ratio) was significantly increased in the T2D group (Figure 2d), compared with the control group (0.9 ± 0.1 vs. 0.6 ± 0.1, *p* < 0.05), and a strong trend remained also after controlling for HbA1c (*p* = 0.052).

### 3.3. Increased F-GLP-1 Is Associated with a Th2 Inflammatory Profile in T2D Patients

**Figure 3:**
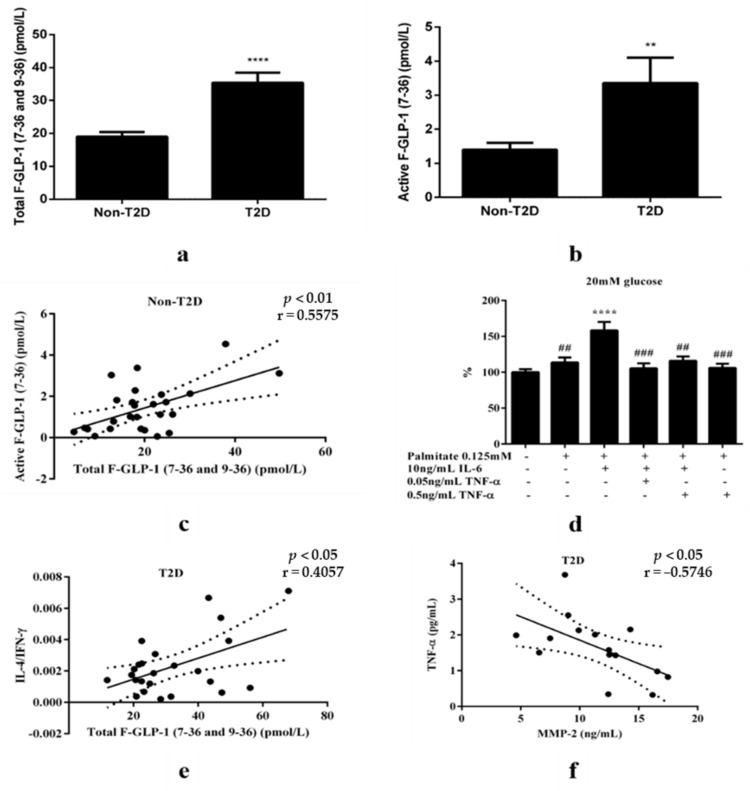
Increased F-GLP-1 is associated with a Th2 inflammatory profile in T2D patients. (**a**) Total F-GLP-1 levels (including GLP-1 7-36 amide and 7-37 [active form] and the metabolite GLP-1 9-36) were higher in T2D patients, as compared to controls, *p* < 0.0001 (*n* = 63 for control, *n* = 37 for T2D). (**b**) Active F-GLP-1 levels were higher in T2D patients, *p* < 0.01 (*n* = 28 for non-T2D, *n* = 8 for T2D). (**c**) A positive correlation was observed between total and active GLP-1 for non-T2D (*n* = 26). (**d**) In the presence of a simulated diabetic milieu (0.125 mM palmitate and 20 mM glucose), IL-6 stimulated GLP-1 secretion is abolished by TNF-α. + Presence of the indicated treatment − absence of indicated treatment. (**e**) A positive correlation between IL-4/IFN-γ ratio and F-GLP-1 levels (*n* = 25) was detected in T2D patients. (**f**) A negative association with TNF-α was observed in the T2D group. Black dots represent analyzed levels for individual samples. Comparisons between groups were performed with an unpaired *t*-test or one-way ANOVA when comparing more than two groups. Significant correlations were assessed with the Pearson correlation coefficient, ** *p* < 0.01; **** *p* < 0.0001. For the in vitro experiment, **** *p* < 0.0001 compared with palmitate and glucose, ## *p* < 0.01, ### *p* < 0.001 compared with IL-6 in the presence of palmitate and glucose.

In accordance with previous results [10], total F-GLP-1 (35.4 ± 3.1 vs. 19.0 ± 1.4 pmol/L *p* < 0.0001, Figure 3a) and active F-GLP-1 (3.4 ± 0.7 vs. 1.4 ± 0.2 pmol/L, *p* < 0.01, Figure 3b) were higher in the T2D group, compared with the control group. As expected, a significant correlation between active and total F-GLP-1 was detected (r = 0.5575, *p* < 0.01, Figure 3c). To exclude potential differences in terms of GLP-1 degradation, we proceeded with an analysis of total F-GLP-1 for the remainder of the study. In vitro studies indicated that IL-6-enhanced secretion from GLP-1 secreting cells was abolished in the presence of TNF-α (Figure 3d, and Supplementary Materials, Figure S1), rendering the elevated IL-6/TNF-α ratio a possible contributor to elevated F-GLP-1. However, no significant correlation was detected between F-GLP-1 and the IL-6/TNF-α ratio (Supplementary Materials, Figure S2), and T2D remained associated with an increased IL-6/TNF-α ratio after controlling for F-GLP-1 (*p* < 0.001). Interestingly though, a significant positive correlation between F-GLP-1 and the relative IL-4/IFN-γ expression (representing a Th2 shift [17]) was detected in the T2D group (r = 0.4057, *p* < 0.05, Figure 3e), and remained after controlling for HbA1c (r = 0.501, *p* < 0.05). Altered plasma MMP-2 activity was not associated with T2D (Supplementary Materials, Figure S3A) or F-GLP-1 (Supplementary Materials, Figure S3B,C, respectively), although a negative association with TNF-α was observed in the T2D group (r = −0.5746, *p* < 0.05, Figure 3f).

### 3.4. Th2 Cytokines Are Downregulated in Patients with TAA

**Figure 4:**
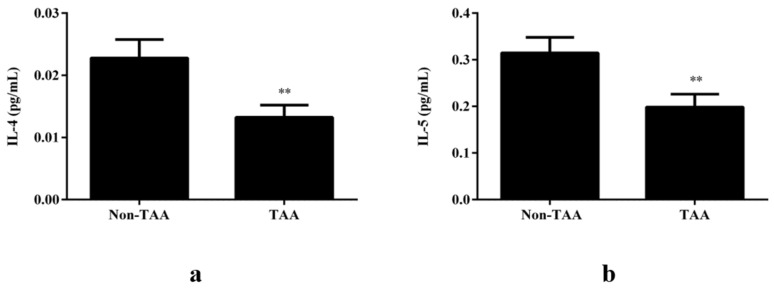
Th2 cytokines are downregulated in patients with TAA. (**a**) IL-4 and (**b**) IL-5 were significantly decreased in the TAA patients, compared with control patients, ** *p* < 0.01 (non-TAA patients; *n* = 49, TAA; *n* = 41). Comparisons between groups were performed with an unpaired *t*-test.

The average aortic diameter for the TAA patient group was 54.4 ± 1.0 mm, compared to the control group with an average aortic diameter of 32.5 ± 0.6 mm. To assess the relevance of the indicated Th2 bias associated with T2D for reduced prevalence of TAA in T2D, we assessed the inflammatory profile of patients with TAAs (Supplementary Materials, Table S2). Although no change was observed in the Th1 cytokines in the TAA group (Supplementary Materials, Table S3), lower plasma levels of the Th2 cytokines IL-4 (0.013 ± 0.002 vs. 0.023 ± 0.003 pg/mL, *p* < 0.01, Figure 4a), and IL-5 (0.20 ± 0.03 vs. 0.31 ± 0.03 pg/mL, *p* < 0.01, Figure 4b) were detected in the TAA patient group, as compared to the control group.

## 4. Discussion

Enhanced GLP-1 signaling due to treatment and/or pathology may contribute to a reduced prevalence of TAAs in T2D, possibly through regulation of inflammatory responses and/or proteolytic activity [18]. Therefore, we determined potential T2D-associated changes in systemic cytokine expression and MMP-2 activity, as well as potential correlations to elevated F-GLP-1. Furthermore, we assessed the relevance of any of the observed changes in the above-mentioned factors in relation to TAAs.

F-GLP-1 levels were increased in T2D patients, which is in agreement with our previous report [10]. Although many studies show reduced postprandial plasma GLP-1 levels in T2D [19], elevated concentrations of F-GLP-1 have also been reported in other patient groups [20]. Altered plasma MMP-2 activity was not associated with T2D or F-GLP-1 despite reports of reduced total plasma MMP-2 in T2D [21]. However, it should be considered that a decrease in total MMP-2 levels does not equate to decreased MMP-2 activity, and the results presented here do not rule out a role for altered MMP-2 activity in the aortic wall—perhaps modulated by GLP-1 signaling—in the reduced prevalence of TAAs in T2D. The negative correlation between TNF-α and MMP-2 activity among T2D patients detected here may implicate that increased insulin resistance is associated with reduced MMP-2 activity.

The fact that T2D was not associated with a significant change in hsCRP or any of the analyzed Th1/Th2 cytokines may seem contradictory, as T2D and obesity are associated with systemic inflammation and enhanced levels of cytokines such as IL-6 [22,23]. However, the control group per se, consisting of non-T2D patients with aortic valve pathology, was characterized by hsCRP levels corresponding to metabolic inflammation. Interestingly though, the increased IL-6/TNF-α ratio in the T2D group, indicates a Th2 bias of immune responses, as IL-6 has well-known Th2 polarizing properties. This Th2 bias in patients with T2D is of interest as an acceleration in the formation/development of AAAs is associated with a Th1 bias [24,25,26]. Further, the strong trend towards reduced plasma levels of IFN-γ in association with T2D agrees with a Th2 bias of immune responses and is of interest considering the association of IFN-γ with an intimal expansion of ascending TAAs [27], and reduced risk of hospitalization for AA among patients with T2D compared with controls [5]. Results from conducted in vitro studies indicate that the increased IL-6/TNF-α ratio may contribute to the elevated F-GLP-1 levels in the T2D group. However, we did not detect a significant association between F-GLP-1 and the IL-6/TNF-α ratio in plasma.

On the other hand, we did detect a significant positive association between F-GLP-1 and the IL-4/IFN-γ ratio, that remained after controlling for HbA1c, indicating that F-GLP-1 levels rise in association with a Th2 shift. Although IFN-γ may act to reduce GLP-1 secretion [28], it is unlikely that the detected association results from effects of IL-4/IFN-γ on the endogenous secretion of GLP-1, particularly considering the lack of an association between F-GLP-1 and IFN-γ. The present study cannot exclude that the association stems from some factor exerting independent effects on systemic inflammation and F-GLP-1. However, it may be that GLP-1R activation on T lymphocytes [29] shapes immune responses, promoting a Th2 shift. GLP-1R activation has previously been shown to promote Th2 responses through upregulation of sirtuin 6 (SIRT6) [30]. However, this remains purely speculative, and future studies should be performed to confirm these associations and unravel underlying mechanisms.

In line with previous reports of an association between AA and Th1 polarized immune responses, we report a Th1 polarization with significantly lower plasma levels of the Th2 cytokines, IL-4, and IL-5, in association with TAA.

Of note, the T2D associated Th2 bias of immune responses detected herein indicates an anti-inflammatory profile that may thus be favorable and prevent/delay TAA formation among patients with T2D. Importantly, systemic inflammation is indicated to have a role in vascular remodeling and future studies should also assess potential associations between altered systemic inflammation in T2D and gene expression changes at the level of the arterial wall. Likewise, potential associations between altered F-GLP-1/the use of GLP-1 receptor analogs/DPP-4 inhibitors and arterial wall gene expression changes associated with TAA should be investigated. Limitations of this study include the lack of discrimination between aortic valve pathologies in the groups (i.e., aortic insufficiency/aortic stenosis). In addition, no information regarding T2D duration, quality, and efficiency of T2D management was available for this study. Furthermore, due to lack of information, smoking status, diet, and physical activity were not accounted for in the analysis or as inclusion/exclusion criteria.

Although the concentration of most cytokines measured was within the expected ranges [31,32], patient characteristics, methodology, and difficulties with the reliability of absolute values measured must be considered. Substantial differences in detected levels of analytes are often found when ELISA kits from different manufacturers are used [33,34,35]. Importantly, we sought to determine whether elevated F-GLP-1 (contributed to by T2D pathology and/or antidiabetic therapy) is associated with an altered inflammatory profile. Therefore, we did not remove T2D patients on antidiabetic therapy that may modulate F-GLP-1. However, it is possible that statins (and/or other medications) contribute to the observed altered systemic inflammation in T2D.

Nevertheless, we report novel findings supporting a Th2 shift of immune responses in T2D patients with aortic valve pathology, as compared to control patients, and a positive association between a rise in F-GLP-1 levels in T2D and the Th2 shift. Associations that appear independent of hyperglycemia. By further confirming an inflammatory profile in TAA while presenting this evidence for an anti-inflammatory Th2 shift in T2D, this study provides information of importance in trying to understand the reduced prevalence of TAA in T2D.

Studies aiming to determine whether antidiabetic therapy using GLP-1 receptor analogs/DPP-4 inhibitors are associated with an altered prevalence of TAAs and altered systemic inflammation/proteolytic activity are currently in the planning stage.

In summary, considering the indicated role for a Th1 inflammatory profile in the development of TAAs, the Th2 shift associated with T2D, potentially contributed to by elevated plasma levels of the gut hormone GLP-1, may play a role in the reduced prevalence of TAAs in patients with T2D.

## Data Availability

The main data supporting the results of this study are presented in this paper or in the Supplementary Materials. The amount of data generated for this study was quite large to be shared publicly, but the raw data can be shared under a reasonable request.

## Supplementary Materials

The following are available online at https://www.mdpi.com/article/10.3390/jcdd8110143/s1, Figure S1: TNF-α does not affect the viability of GLUTag cells, Figure S2: F-GLP-1 does not correlate with IL-6/TNF-α ratio in the T2D group, Figure S3A–C: Systemic MMP-2 activity is not associated with T2D nor with F-GLP-1. Table S1: Patient characteristics, Table S2: T2D was not associated with a significant change in hsCRP or any of the other cytokines measured. Table S3: Levels of the inflammatory markers in TAA.
