# Supplementary material for: Elevated Glucagon-like Peptide-1 and a Th2 Shift May Support Reduced Prevalence of Thoracic Aortic Aneurysm in Patients with Diabetes
Source: J Cardiovasc Dev Dis. 2021 Oct 28;8(11):143. doi: 10.3390/jcdd8110143 (PMC8618274; doi:10.3390/jcdd8110143)
Supplement: Supplementary file 1 [file jcdd-08-00143-s001.zip › jcdd-1421403-supplementary.pdf]

Journal of
Cardiovascular Development and Disease

MDPI

SUPPLEMENTARY MATERIAL

Expanded Material and Methods

GLP-1 Secretion Studies

GLUTag cells are an immortalized cell line secreting the GLP-1 peptide. The medium was supplemented with 10% FBS (Thermofisher Scientific, Waltham, Massachusetts), 5.5 Mm glucose (Sigma- Aldrich, St.Louis, Missouri), 10,000 U/mL penicillin and 10,000 μg/ML streptomycin sulphate (Thermo Fisher Scientific, Waltham, Massachusetts). Palmitate was used to stimulate diabetic hyperlipidemia and it was dissolved in 50% ethanol at 70 °C to create a 100 mM stock. Control cells were given a vehicle with equal amounts of ethanol as the palmitate exposed cells (final concentration of ethanol: 0.12%).

GLUTag cells were plated in a 6-well plate at a density of 250,000 cells/mL and incubated with/without 0.125 mM palmitate, in the presence/absence of 10 ng/ml IL-6 and / or 0.05/0.5 ng/mL TNF-α (as indicated in Figure 3) concentrations for 48 h. After 48 h, cells were washed in Krebs-Ringer bicarbonate HEPES buffer (KRBH) with 0.2% Bovine serum albumin (BSA) containing 0.5 mM glucose. After a subsequent 30 min pre incubation in the same type of buffer, cells were exposed to 20 mM glucose in KRBH/0.2% BSA buffer for 1 h. Total GLP-1 in the incubation buffer after 1 h was determined using an ELISA kit (Cat. No. EZGLP1T 36K, Millipore, Burlington Massachusetts) according to the manufacturer's instructions.

Caspase 3 Activity Assay

GLUTag cells were plated and cultured for 24 h in a 6-well plate at a density of 180,000 cells/mL. The cells were incubated with/without palmitate/IL-6/TNF-α (indicated concentrations) for 48 h. Caspase-3 activity was determined using an assay kit (Cell Signaling Technology) according to the manufacturer's instructions. Briefly, the colorimetric assay used is based on the hydrolysis of a caspase-3 substrate generating a fluorescent product, which can be detected at excitation/emission 342/441 nm. Results from the caspase-3 activity assay were normalized by the total protein concentration by using the DC protein assay (Bio-Rad Laboratories, Hercules, California).

Tables

Table S1. Patient characteristics. Age, anthropometry, anti-diabetic medications, aortic valve pathology, and gender distribution of individual groups.

	Non-T2D Non-TAA	T2D	TAA	Total Participants
Number of participants	65	37	42	144
Mean Age (± SEM)	68.3 ± 1.6	72.0 ± 0.86	65.5 ±1.7	68.5 ± 0.9
Gender (Male/Female)	38/27	29/8	26/16	93/51
Valve pathology (AS/AI)	38/21	34/1	3/30	75/52
Insulin		4		
Metformin		16		
Sulfonylurea or meglitinides		2		
Metformin and insulin		2		
Metformin and sulfonylurea or meglitinides		3		
Metformin and DPP-4 inhibitor		3		

Insulin and DPP-4 inhibitor	1
Metformin and sulfonylurea and insulin	1
No medication	5

Table S2. T2D was not associated with a significant change in hsCRP or any of the other **cytokines measured**. For hsCRP, n = 67 for non-T2D, n = 35 for T2D; for cytokines, n = 49 for non-T2D, n = 33 for T2D.

	**Non-T2D**	**T2D**	***p*-Value**
Systolic blood Pressure (mmHg)	143.3 ± 2.5	141.9 ± 3.2	0.7
Diastolic blood Pressure (mmHg)	77.1 ± 1.7	79.3 ± 1.9	0.4
hsCRP (mg/L)	1.93 ± 0.22	1.69 ± 0.27	0.5
IFN-γ (pg/mL)	10.02 ± 1.10	7.96 ± 0.86	0.06
IL-1β (pg/mL)	0.04 ± 0.005	0.04 ± 0.006	0.86
IL-4 (pg/mL)	0.02 ± 0.003	0.03 ± 0.004	0.48
IL-5 (pg/mL)	0.31 ± 0.03	0.24 ± 0.03	0.11
IL-6 (pg/mL)	1.17 ± 0.12	1.09 ± 0.12	0.61
IL-12p70 (pg/mL)	0.16 ± 0.02	0.16 ± 0.02	0.95
TNF-α (pg/mL)	1.58 ± 0.13	1.66 ± 0.13	0.66

Table S3. Levels of the inflammatory markers in TAA. For hsCRP n= 67 for non-TAA, n = 44 for TAA; for cytokines n = 49 for non-TAA, n = 41 for TAA**.**

	**Non-TAA**	**TAA**	***p* Value**
hsCRP (mg/L)	1.93 ± 0.22	2.39 ± 0.33	0.25
IFN-γ (pg/mL)	10.02 ± 1.10	7.48 ± 0.81	0.07
IL-1β (pg/mL)	0.04 ± 0.005	0.05 ± 0.008	0.42
IL-4 (pg/mL)	0.02 ± 0.003	0.01 ± 0.002	< 0.01
IL-5 (pg/mL)	0.31 ± 0.03	0.20 ± 0.03	< 0.01
IL-6 (pg/mL)	1.17 ± 0.12	0.97 ± 0.12	0.22
IL-12p70 (pg/mL)	0.16 ± 0.02	0.13 ± 0.01	0.15
TNF-α (pg/mL)	1.58 ± 0.13	1.41 ± 0.10	0.32

Figures

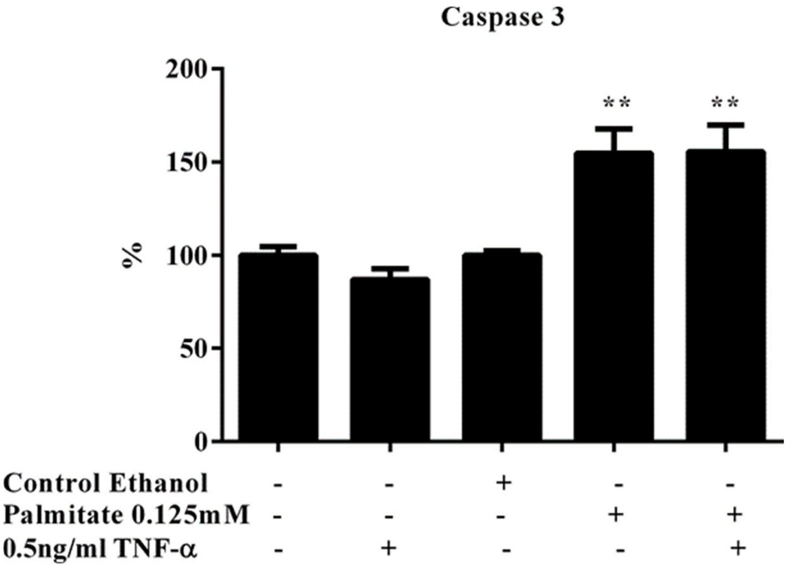

Figure S1. TNF-α does not affect the viability of GLUTag cells. Caspase-3 activity was used as a measure of induced cell death following TNF-α exposure in the presence/absence of palmitate. The concentration of TNF-α used to block the effect of IL-6 on glucose-stimulated-GLP-1 secretion (0.5 ng/mL) did not significantly alter caspase-3 activity in GLP-1-secreting GLUTag cells (in vitro experiment). The presence of simulated diabetic hyperlipidemia (palmitate exposure) did not alter this finding. All the experiments were run in duplicate and repeated four times. Comparisons between groups were performed with a one-way ANOVA, ** $p < 0.01$ compared with appropriate control. + Presence of the indicated treatment. − absence of indicated treatment.

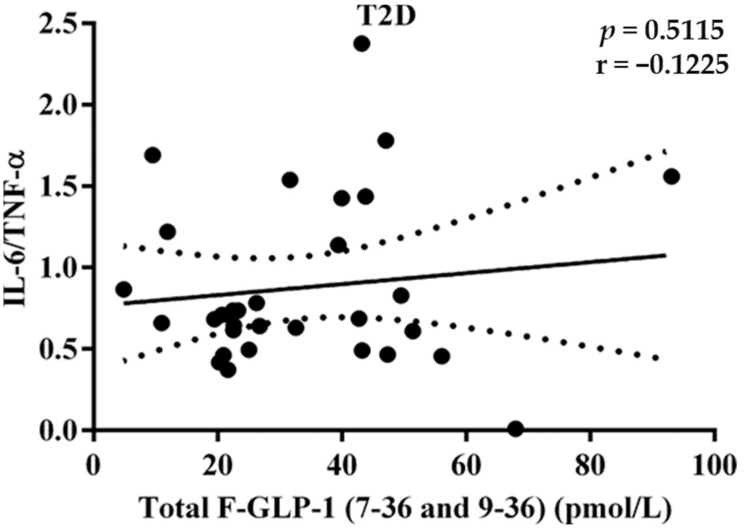

Figure S2. F-GLP-1 does not correlate with IL-6/TNF-α ratio in the T2D group. The correlation was assessed using the Pearson correlation coefficient, $n = 26$ for T2D. Black dots represent analyzed levels for individual samples.

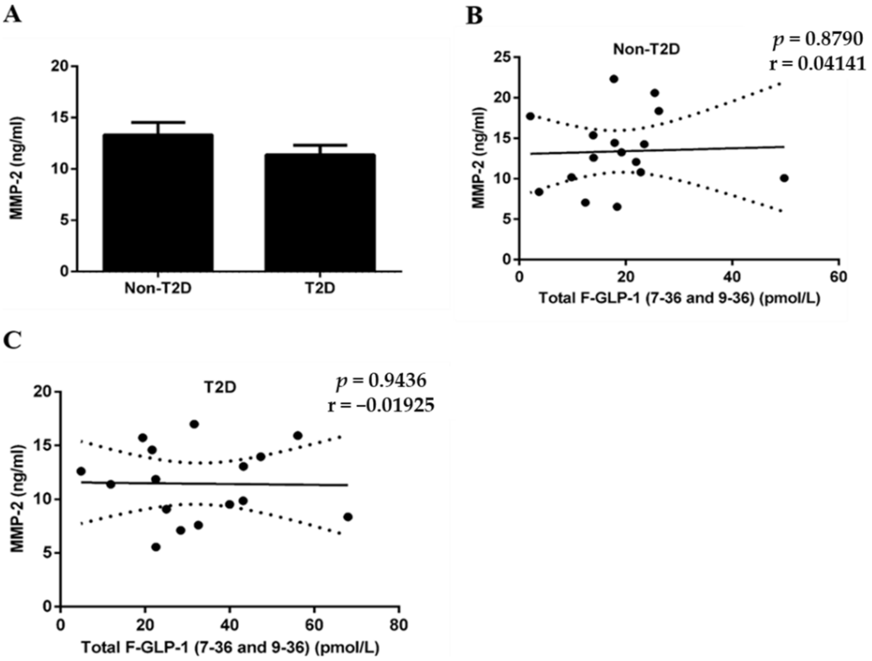

Figure S3. Systemic MMP-2 activity is not associated with T2D nor with F-GLP-1. (**A**) Plasma MMP-2 activity was slightly, but not significantly, reduced in the T2D group compared with controls. Furthermore, F-GLP-1 levels were not indicated to influence MMP-2 activity, as no correlation between plasma GLP-1 and MMP-2 activity was detected in patients (**B**) without or (**C**) with T2D. Comparisons between groups were performed with an unpaired t-test. Correlations were assessed using the Pearson correlation coefficient, n = 17 for non-T2D (controls) and n = 16 for T2D. Black dots represent analyzed levels for individual samples.
